# Do members of cancer peer support groups know more about cancer than non-members? Results from a cross-sectional study in Germany

**DOI:** 10.1007/s00520-022-07488-3

**Published:** 2022-12-13

**Authors:** Elâ Ziegler, Jens Klein, Christopher Kofahl

**Affiliations:** Institute of Medical Sociology, University Medical Center Hamburg-Eppendorf, Martinistraße 52, 20246 Hamburg, Germany

**Keywords:** Oncology, Cancer, Knowledge, Health literacy, Peer support, Self-help groups

## Abstract

**Purpose:**

This study aims to assess whether cancer-specific knowledge (CSK) is associated with membership in a cancer peer support group (PSG) and other factors.

**Methods:**

A cross-sectional study investigated the CSK of 1121 cancer patients of various entities across Germany. CSK was measured with the BCKS-14, a 14-item knowledge instrument which was previously participatory developed with patient representatives and oncologists. Associations between CSK and PSG membership, sociodemographic factors, internet use, and preferences in medical decision-making were analysed with *t*-tests and multiple linear regressions.

**Results:**

The *t*-test showed a statistically significant difference in CSK between members and non-members of PSGs. Knowledge for PSG members was on average 0.97 points higher (*p* < 0.001) and varied between 2 and 14 points compared to 0–14 points for non-members. Regression analysis revealed age, gender, time since diagnosis, education, internet use, and PSG activity to be statistically significant predictors. Younger (*β* =  − 0.15; *p* < 0.001), female (*β* = 0.10; *p* = 0.001), higher educated patients (*β* = 0.27; *p* < 0.001) with and a diagnosis longer ago (β = 0.10; p = 0.002) who use the internet frequently for information seeking (*β* = 0.20; *p* ≤ 0.001) and members of cancer PSGs (*β* = 0.18; *p* ≤ 0.001) showed a higher CSK.

**Conclusion:**

Overall, CSK of the participants shows a high degree of variance. CSK should be promoted for all patients and especially for older, newly diagnosed patients with low educational levels and PSGs introduced early on as they contribute to improving CSK among other benefits.

**Supplementary Information:**

The online version contains supplementary material available at 10.1007/s00520-022-07488-3.

## Introduction

Disease-specific knowledge is a core component of cancer patients’ health literacy and thus highly relevant for making informed decisions [1]. It is associated with effective self-management [2] and can consequently improve physical and mental outcomes [3–6]. As a result, it can foster empowerment of patients [7]. However, studies indicate that many cancer patients lack cancer-specific knowledge [4, 8–10], or that patients feel like they do not know enough about the complex treatment options, side effects, and the care system [11]. This in turn has implications for patients’ decision-making and the course of the disease [10].

Cancer peer support groups (PSGs) have the potential to improve patients’ disease-specific knowledge through shared informational support [12, 13]. Cancer peer support provided in groups of individuals with the same disease who meet outside professional settings and without hierarchical relationships has therefore become a crucial part of effective supportive oncological care. With regard to who is joining a PSG, several studies have shown that patient characteristics such as age, gender, socioeconomic status, education, disease duration, and social support have an influence on the decision whether to participate in a PSG or not [14–18]. However, studies assessing the predictive value of these factors relating to participation in PSGs produced ambiguous results [14–18]. Concerning cancer-related knowledge, studies similarly identified especially female and elderly patients, and those with lower education to be at risk for having insufficient knowledge to effectively manage their disease [4, 6, 19].

To date, it has not yet been sufficiently studied how much patients inside and outside of PSGs know about cancer. Next to some qualitative studies on this topic [20, 21], there are only few quantitative studies comparing cancer-related knowledge between PSG members and non-members and have various limitations. Most of these studies examine only one gender-specific entity like breast or prostate cancer [8, 22–27], so that differences due to gender are not assessed and have only small sample sizes [22, 28]. Further, the instruments used in these studies are often subjective self-assessments [22, 23, 28–30] rather than objective knowledge tests and not validated [25, 30]. Lastly, the existing instruments were not developed with patients for patients and may therefore not be appropriate for all cancer patients.

Against this backdrop, we use a newly, participatory developed brief cancer knowledge scale (BCKS-14). It comprises cancer-specific knowledge content considered as relevant for patients of all cancer entities from the view of patients. We aim to assess firstly, how cancer-related knowledge is associated with participation in PSGs to investigate whether members of PSGs know more about cancer than non-members. Secondly, we explore which other factors are associated with higher or lower cancer-specific knowledge.

## Methods

### Study design and population

A cross-sectional study with a self-administered questionnaire was conducted between October 2020 and September 2021 to examine cancer-related knowledge among cancer patients across Germany. The research is part of a larger study investigating health literacy, self-help activities, and care experiences of cancer patients. The study was based on a participatory research approach and conducted in cooperation with the House of Cancer Self-Help–Federal Association (HCSH), an association of ten nationwide operating cancer peer support organisations (PSOs) funded by the German Cancer Aid Foundation. Ethical approval was obtained from the Local Psychological Ethics Committee at the Centre for Psychosocial Medicine, University Medical Centre Hamburg (No. LPEK-0109).

Recruitment of patients commenced in October 2020, following a multi-channel approach sending more than 60,000 pamphlets and posters containing study information to over 1382 cancer care facilities for acute, supportive, and after care across Germany. The study material provided a link and QR code to participate in the survey online as well as a telephone number and e-mail address for patients who might wish to use a paper–pencil version of the questionnaire, which was then sent to them by post and could be returned anonymously. The care and counselling facilities such as regional cancer societies, cancer counselling centres, oncological rehabilitation clinics, certified cancer centres, hospitals with oncological departments, and oncological specialised practices as well as peer support organisations were informed in advance about the study by post.

Snowball sampling of patients also took place though the HCSH, peer support groups outside of PSOs, and the German Cancer Society, asking them to circulate the call for study participation (by e-mail, post and PSO-journals). Further, information about the study was shared through a newsletter of the National Contact and Information Centre for the Initiation and Support of Self-Help Groups and at a virtual patient congress. Reminder e-mails were sent out in February and May 2021 to optimise response rates. Eligible participants were German-speaking members and non-members of PSGs that are patients 18 years and older, with any cancer diagnosis at any stage, regardless of gender and treatment received. All participants provided informed consent by confirming to have read the study information and data protection regulations.

### Instruments and variables

Data for this study are collected using a self-administered questionnaire which is part of a larger questionnaire focussing on nine topics: diagnosis and treatment, care experience, self-help activity, health literacy, coping and self-management, social support and quality of life, economic situation, religiosity/spirituality, Covid-19, and sociodemographic information. The survey questionnaire was created in collaboration with medical representatives of oncology, representatives of medical sociology, the German Cancer Society, members of HCHS and PSOs, peer support researchers, and self-help clearing houses.

#### Outcome variable

Cancer-related knowledge as the independent variable was measured using a 14-item questionnaire (BCKS-14) (see Appendix) previously developed by the authors. It represents the extended version of the BCKS-10 which was validated in a sample of 500 cancer patients and showed satisfactory internal consistency (Cronbach’s *α* = 0.68) [31]. The BCKS-14 contains four additional nation-specific items about German (social) legislation and patient rights and showed similar psychometric properties (Cronbach’s *α* = 0.68). The brief cancer knowledge scale includes cancer-specific elements of knowledge about terminology, diagnosis, treatment, (social) legislation, and numeracy that were identified as crucial for patients in a previous study by PSG leaders. For analyses, we coded the correct answers as ‘1’ and both the incorrect and the ‘don't know’ answers as ‘0’ and built a sum score ranging from 0 to 14 points. Thus, patients received 1 point per correct answer and could reach a maximum of 14 points if all answers were answered correctly (see Appendix). We accepted up to three missing answers, which were imputed for building the sum score, so if more than three answers were missing, the respondent was counted as missing.

#### Independent variables

To measure patients’ preference regarding medical decision-making, we used a modified version of item 13 of the Patient Participation Questionnaire [32] in order to assess their general attitude towards different models of decision-making. Patients were asked to indicate who should make the medical decisions for their disease, ranging between 1 (active, informed decision-making model preference; ‘I should decide’.) and 5 (equivalent to passive, paternalistic model preference; ‘The doctor should decide’,). To assess internet use for cancer information, patients were asked to position themselves on a 4-point scale to indicate how intensively they have used information from the internet to inform themselves about cancer, ranging from 1 ‘almost exclusively’ to 4 ‘not at all’. For the analyses, values for decision-making preference and internet use were reversed so that the scales range from passive/less frequent to active/more often.

Other predictors comprised patient sociodemographic characteristics (gender, age, education, and relationship status as a proxy for social support), clinical history (time since diagnosis) as well as PSG activity (membership). PSG membership was dichotomised into ‘currently a member of a PSG’ and ‘never been a member of a PSG’. Partnership status was also dichotomised into having a spouse or partner/not having a spouse or partner. Age and time since diagnosis were coded in years as continuous variables. School education was coded into high, medium, and low, representing ≤ 9 years of education (no qualification or lower secondary school leaving certificate, *Hauptschulabschluss*), 10 years of education (*Realschulabschluss*), and ≥ 11 years (*Fachhochschulreife/Abitur*) level of education.

### Statistical analyses

Data analysis was performed using IBM SPSS Statistics 26. Descriptive statistics were used to examine clinical and sociodemographic characteristics of patients and the distribution of scores of the outcome variables. Two-tailed independent *t*-test was used to evaluate the difference in knowledge scores between PSG members and non-members adjusting for multiple testing according to Holm’s procedure [33]. Multiple linear regression was used to determine potential associations between knowledge scores, PSG membership, time since diagnosis, internet use, decision-making preference, and sociodemographic variables such as gender, age, education, and partnership. The statistical significance was set to an alpha level of 0.05.

## Results

### Sample characteristics

A total of 1356 patients participated in the study. We excluded respondents with missing data for items regarding the sociodemographic characteristics. After data cleaning, 1121 patients who completed the questionnaire remained in the data set. Table 1 summarises the characteristics of these respondents. Patients from all federal states of Germany participated, most of them from North Rhine-Westphalia, the federal state with the biggest population in Germany. Newly diagnosed patients as well as cancer survivors participated, on average 4.6 ± 6.0 years after the cancer diagnosis. The mean age was 61.3 ± 12.4 years. The percentage of female participants was 54.7%. Most respondents had a high level of education (58.3%), which is almost twice as high as German general population in 2018 [34] and were living in a partnership (83.2%), compared to 60% in the general population [35]. Nearly a third of the respondents were breast cancer patients (30.6%) followed by prostate cancer patients as the second most common cancer type (19.3%). The cancer stages varied from UICC (Union Internationale Contre le Cancer) stage 0 to stage IV, with most patients not knowing their stage of the disease (43.5%).

**Table 1:** Characteristics of the patients (*N* = 1121)

Variable	Patients, *n* (%) or mean (SD)	PSG-members, *n* (%) or mean (SD)	Non-members, *n* (%) or mean (SD)	*p*
Age (years)	61.3 (± 12.4)	65.7 (± 11.5)	57.5 (± 11.9)	**.000**^**a**^
21–39	66 (5.9%)	19 (3.8%)	46 (7.7%)	
40–59	400 (35.7%)	109 (22.1%)	285 (47.5%)	
60–79	589 (52.5%)	320 (64.8%)	251 (41.8%)	
≥ 80	66 (5.9%)	46 (9.3%)	18 (3.0%)	
Gender				**.000**^**b**^
Male	507 (45.3%)	304 (61.5%)	188 (31.4%)	
Female	613 (54.7%)	190 (38.5%)	411 (68.6%)	
Education				.442^b^
Low (≤ 9 years)	135 (12.2%)	59 (12.0%)	74 (12.6%)	
Medium (10 years)	326 (29.5%)	138 (28.1%)	184 (31.3%)	
High (≥ 11 years)	645 (58.3%)	294 (59.9%)	330 (56.1%)	
Partnership				**.048**^**b**^
No	184 (16.8)	69 (14.2%)	109 (18.8%)	
Yes	909 (83.2%)	416 (84.2%)	472 (81.2%)	
Primary cancer type				**.000**^**b**^
Breast cancer	337 (30.6%)	89 (18.1%)	243 (41.7%)	
Prostate cancer	212 (19.3%)	151 (30.6%)	55 (9.4%)	
Bladder cancer	91 (8.3%)	72 (14.6%)	15 (2.6%)	
Colorectal cancer	77 (7.0%)	37 (7.5%)	39 (6.7%)	
Leukaemia	39 (3.5%)	20 (4.1%)	19 (3.3%)	
Lymphoma	37 (3.4%)	13 (2.6%)	22 (3.8%)	
Lung cancer	32 (2.9%)	10 (2.0%)	22 (3.8%)	
Bone marrow cancer	28 (2.5%)	13 (2.6%)	14 (2.4%)	
Thyroid cancer	28 (2.5%)	14 (2.8%)	13 (2.2%)	
Skin cancer	24 (2.2%)	10 (2.0%)	14 (2.4%)	
Laryngeal cancer	23 (2.1%)	18 (3.7%)	4 (0.7%)	
Kidney cancer	22 (2.0%)	4 (0.8%)	18 (3.1%)	
Other (overall each less than 2%)	150 (14.2%)	42 (8.5%)	119 (20.4%)	
UICC stage				**.032**^**a**^
0	16 (1.5%)	8 (1.7%)	7 (1.3%)	
I	114 (11.0%)	47 (10.1%)	64 (11.7%)	
II	160 (15.4%)	75 (16.1%)	83 (15.2%)	
III	210 (20.3%)	101 (21.7%)	101 (18.5%)	
IV	86 (8.3%)	52 (11.2%)	34 (6.2%)	
do not know	451 (43.5%)	182 (39.1%)	257 (47.1%)	
Time since diagnosis	4.6 (± 6.0)	7.7 (± 6.5)	1.8 (± 3.5)	**.000**^**a**^
≤ 1 year	345 (30.9%)	35 (7.1%)	310 (51.8%)	
1–4 years	370 (33.1%)	153 (31.0%)	210 (35.1%)	
≥ 5 years	403 (36.0%)	305 (61.9%)	78 (13.0%)	
Peer support group membership				
No	600 (54.8%)	0 (0%)	600 (100%)	
Yes	494 (45.2%)	494 (100%)	0 (0%)	
Knowledge score	9.4 (± 2.6)	10.0 (± 2.3)	9.0 (± 2.8)	**.000**^**a**^
Medical decision-making preference				.133^b^
Paternalistic model	69 (6.2%)	23 (4.7%)	45 (7.6%)	
Shared decision-making	841 (76.0%)	373 (76.6%)	448 (75.5%)	
Informed decision-making	197 (17.8%)	91 (18.7%)	100 (16.9%)	
Internet use for information				**.009**^**b**^
Not at all	164 (14.7%)	53 (10.8%)	107 (18.0%)	
Rarely	399 (35.8%)	187 (37.9%)	206 (34.6%)	
Mainly	466 (41.8%)	216 (43.8%)	236 (39.7%)	
Almost exclusively	86 (7.7%)	37 (7.5%)	46 (7.7%)	

Significant differences are highlighted in bold

^a^*t*-test

^b^chi^2^-test

Nearly half of the participants were members of PSGs (45.2%). Their membership reached from less than a year up to 42 years, with an average membership of 15.0 years. Comparing PSG members with non-members, both groups had a similar education status and the decision-making preference. All patients prefer shared-decision-making for medical decisions followed by the informed decision-making model. However, there are significant differences in age, gender, cancer type, and time since diagnosis among the groups, with PSG members being mostly males and older, with a diagnosis on average 6 years longer ago compared to non-members. More PSG members are prostate cancer survivors who completed treatment. More frequent internet use for cancer-related information seeking is slightly higher among PSG members. Further, there are statistically significant differences with minimal effect sizes regarding partnership status and UICC stages but with overall similar distributions across both groups.

### Unpaired t-test for independent samples (Welch’s t-test due to inhomogeneity of variance)

**Fig. 1:**
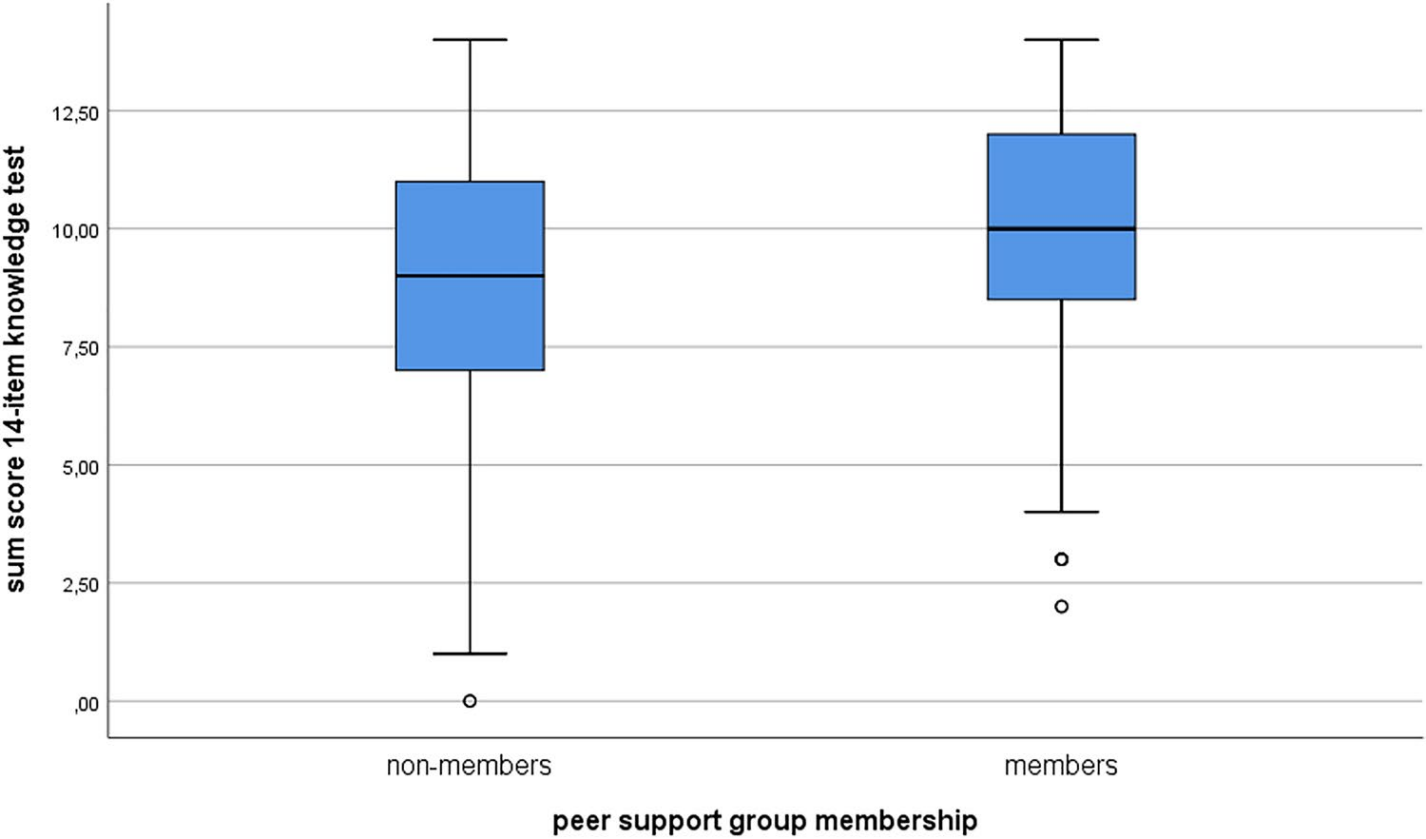
Sum score comparison of knowledge test for members and non-members of peer support groups (*n* = 1094)

A *t*-test was performed to detect meaningful differences between PSG members and non-members in cancer-specific knowledge. Both groups were not normally distributed, as assessed by the Shapiro–Wilk test (*p* < 0.001) and Kolmogorov–Smirnov test. However, due to large sample sizes (*n* > 30) for each of the two groups, normally distributed means were assumed and parametric tests appropriate be used [36, 37]. There were 494 (members) and 600 (non-members) participants with valid data for the knowledge test in the groups (*n* = 1,094). Cancer-specific knowledge scores ranged from 0 to 14 points in non-members and from 2 to 14 points among PSG members. Knowledge sum scores of the 14 items in total were higher among members (*M* = 9.94, *SD* = 2.34) than non-members (*M* = 8.96, *SD* = 2.79) (Fig. 1). The Levene test became significant, and thus, there was no variance homogeneity for the two groups (variances are not equal), so the Welch test statistic was assessed. There was a statistically significant difference between overall knowledge scores of PSG members and non-members, with mean knowledge scores 0.97 points (95%-CI = 0.67–1.28) higher for PSG members, t(1081.84) = 6.25, *p* < 0.001.

Comparison of mean knowledge scores between PSG members and non-members regarding the individual knowledge questions (single items) revealed significant higher scores of PSG members than non-members in 7 out of 14 items (questions 4, 7, 8, 9, 10, 11, and 13). Those differences were however marginal. Besides, PSG members scored marginally worse than non-members on items 6 and 12; however, those differences are not statistically significant. The largest significant differences were visible in questions 8, 9, and 11; each showed a mean score difference of > 0.10) in favour of the PSG members (Table 2).

**Table 2:** Mean score differences (*t*-tests) among peer support group members and non-members (*n* = 1094)

Item	Mean score difference	95%-CI	*p*
Definition of tumour stage I	0.06	0.00–0.11	0.190
Allocation of 80% drug efficacy	0.04	− 0.00–0.09	0.320
Meaning of 5% incidence	0.02	-0.03–0.06	1.000
Aim of palliative care	0.07	0.03–0.11	**0.007**
Calculation of risk reduction	0.05	− 0.01–0.11	0.427
Definition of metastasis	− 0.00	− 0.01–0.01	1.000
Definition of cytostatics	0.10	0.05–0.15	** < 0.001**
Definition of colonoscopy	0.18	0.13–0.23	** < 0.001**
Allocation of false positive result	0.12	0.06–0.18	**0.001**
Definition of adjuvant therapy	0.09	0.03–0.15	**0.020**
Recommended start of follow-up treatment	0.22	0.17–0.28	** < 0.001**
Maximum duration sick pay	− 0.05	− 0.11–0.01	0.427
Application for a disabled person's card	0.07	0.02–0.11	**0.021**
Patients’ rights	0.02	− 0.04–0.08	1.000

Adjusted for multiple testing (Holm’s procedure), significant differences are highlighted in bold

### Multiple linear regression

To determine more comprehensively the association of PSG membership and other factors on cancer-specific knowledge, a multiple linear regression was performed. The regression model includes cancer-related knowledge as the outcome variable and nine explanatory variables as presented in Table 3. Prior to the analysis, we checked for normality, linearity, multicollinearity, and independence of residuals — no concerns were found. Indicating a normal distribution, the analysis revealed the model to be a good fit to the data (*F*(8,1024) = 40.94, *p* < 0.001), being statistically significant and explaining 24% of the variability in the dependent variable (adjusted *R*2 = 0.24). Of the predictor variables, age, gender, time since diagnosis, education, PSG activity, and internet use were found to be statistically significant. Younger (*β* =  − 0.15, *p* ≤ 0.001), female patients (*β* = 0.10, *p* = 0.001) and those with a diagnosis longer ago (*β* = 0.10, *p* = 0.002) were more likely to have higher cancer-related knowledge. Further, the higher the educational level (*β* = 0.27, *p* ≤ 0.001), the higher is the knowledge score. Lastly, members of PSGs and patients who use the internet more for accessing cancer-related information hold higher overall disease-specific knowledge scores (*β* = 0.18, *p* ≤ 0.001 and *β* = 0.20, *p* ≤ 0.001).

**Table 3:** Linear regression model examining cancer-related knowledge (*n* = 1032)

Independent variables	Regression coefficient Β	Standard error	Standardised regression coefficient β	95%-CI	*p*
Age	− 0.031	0.007	− 0.152	− 0.05 to − 0.02	** < 0.001**
Gender	0.535	0.166	0.103	0.21–0.86	**0.001**
Years since diagnosis	0.045	0.014	0.101	0.02–0.07	**0.002**
Education	1.010	0.106	0.273	0.80–1.22	** < 0.001**
Partnership	0.056	0.194	0.008	− 0.33–0.44	0.773
Peer support group membership	0.915	0.168	0.176	0.59–1.24	** < 0.001**
Decision-making preference	0.048	0.111	0.012	− 0.17–0.27	0.666
Internet use	0.640	0.091	0.203	0.46–0.82	** < 0.001**

Significant variables are highlighted in bold

## Discussion

The patient groups representing PSG members and non-members in this study were heterogeneous but similar in terms of education levels, decision-making preference, relationship status, and internet use for information. In both groups, cancer knowledge showed a wide range and overall moderate to high average knowledge scores (*M* = 9.94 among members and 8.96 of non-members). This means, on average, the patients have answered at least 9 out of 14 questions correctly. The overall knowledge levels detected in this study seem to contradict the finding from Fagerlin et al. [8], who found that the majority of recently diagnosed breast cancer patients had generally low knowledge to make informed decisions about breast cancer and treatment options. Yet, this indicates that before and shortly after diagnosis, cancer-relevant knowledge is most likely still low and increases in the further course of the disease and its treatment. This is consistent with our finding that the time since diagnosis correlates highly significantly with the knowledge index.

The present results also show that cancer-specific knowledge was significantly higher among PSG members compared to non-members. Thus, they support the findings of prior research that concluded that PSGs indeed contribute to extended cancer information among their members [24, 25, 27]. However, although the mean difference between the groups in our study was significant, it was rather small. PSG members on average have answered only one more knowledge question correctly than non-members. This raises the question of how far this difference presents a practical relevance. Considering the fifth of a standard deviation as often suggested by the literature to estimate the minimally important difference (MID) with a small effect size [38–40], the 0.97 point difference in our data exceeds the determined MID of 0.53. Thus, we can assume a small effect but meaningful difference for cancer care. This finding is in line with the above cited studies [24, 25, 27], which also found small to moderate but significant effects when comparing cancer PSG members’ with non-members’ knowledge, even after controlling for sociodemographic variables.

Further studies, which did not find any difference between PSG-members and non-members regarding objective cancer knowledge, however found subjectively perceived improvements in knowledge through the PSGs [16, 26]. Similarly, Sheppard et al. [28] reported that PSG participants subjectively felt better informed than non-participants, concluding that PSGs can also foster patients’ empowerment. Overall, these results demonstrate the importance of PSGs, providing not only emotional support but informational support as well. Nevertheless, considering that the questions asked in this study were overall not too challenging as assessed by the item difficulty index of the BCKS [31], the large range of knowledge scores and average sum scores should have been higher among both groups.

Other factors associated with higher levels of cancer knowledge despite PSG activity as revealed in the regression analysis were age, time since diagnosis, gender, education, and internet use. In comparison to the other factors, the association of PSG membership with cancer knowledge was weaker than the association with other factors, as reported previously [25] as well. This might stem from the fact that PSGs are not necessarily systematic training programmes targeting knowledge as the primary aim, but rather provide informational support as one of several other aspects such as emotional support. Yet, it represents the third strongest predictor on knowledge in our regression model (peer support members achieve one point more in the knowledge score, as indicated by the beta values).

The association between educational level and cancer knowledge is the highest compared to the other predictors in our data, as higher educated patients achieve one point more on the knowledge scale, as shown by the beta values. It is not surprising that patients with higher education hold higher disease-specific knowledge and this finding was equally reported previously [6, 25], who also highlight education being a stronger predictor on total knowledge scores than PSG membership. Noeres et al. [27] on the other hand identified PSG involvement as most decisive for participants’ knowledge but also acknowledge the effects of age and education. Thus, it can be concluded that the impact of a PSG on cancer knowledge is an additional stand-alone factor similar to age, disease duration, gender, or internet use. The analysis further revealed that internet use is positively associated with cancer-related knowledge, which reflects the assumption that over the last two decades, the internet has become a meaningful source for cancer information and thus cancer knowledge. Here, the standardised beta values indicated internet use to be the second strongest predictor (achieving more than 0.5 points more on the knowledge scale, if used more frequently). This result supports the findings of other authors [4, 8], who found that internet use or daily internet access improves cancer knowledge.

Interestingly, a steady relationship as an indicator of social support did not predict cancer knowledge, while other studies did find social support to have an impact [41]. Our finding may suggest that the patients’ partners are not well informed either or that they have not been sufficiently involved in the patients’ cancer history. Nonetheless, further studies have shown that including partners or relatives in treatment and care indeed improves communication and patients’ compliance as they often have more capacities to absorb and remember relevant disease-specific information than the patients themselves, specifically in their overwhelming situation [42, 43]. As shown in our analyses, older patients tend to hold less disease-specific knowledge, possibly due to lack of recall of information and effective communication with health care providers [44] and because they might be less likely to effectively obtain information from the internet. Especially for them, it can be helpful, and thus is to be recommended, to include relatives in information provision and also to suggest participation in a PSG to help strengthen cancer-specific knowledge.

Contrary to previous research [6] reporting higher health literacy scores among male patients, female gender predicted a higher cancer knowledge in our study. Here, the association with knowledge was weaker than other predictors, and being female led to achieving merely half a point more on the knowledge test. Moreover, the medical decision-making preference was not significantly associated with cancer knowledge. Thus, the results could not confirm that those patients who prefer an active involvement in medical decision-making with their physicians have higher knowledge scores, although the patients in this sample had overall satisfactory knowledge and preferred a rather active role.

While earlier work from Kühner et al. [25] did not find duration of the disease to have an effect on knowledge levels, we found newly diagnosed patients to hold lower cancer-specific knowledge than those with a diagnosis longer ago. This indicates that in a phase of orientation and acute treatment immediately after the diagnosis, patients are often not able to process all information presented by physicians and others [43] and it may first be a matter of sheer survival. More questions about the disease and its long-term effects such as probability of recurrence, (socio-) legal matters, or dealing with disability only come into focus in the longer run after the first treatment phase or as the patients return to their everyday lives. Our findings therefore indicate that cancer survivors become experts of their illness over time by gathering more information about the disease step by step. Lastly, it is usually not directly after diagnosis but at a later point in time that patients join a PSG, as shown by the patients’ characteristics in this study and as revealed by Stevinson et al. [14] which underlines the effect of time since diagnosis on the development of knowledge. Yet, as PSGs have shown to have the potential to improve cancer knowledge, which in turn may impact treatment decision-making, early access to PSGs could be valuable in order to ensure early access to critical cancer-related information.

### Limitations

Though the findings provide some evidence for how cancer-related knowledge is associated with participation in PSGs and what other factors are associated with cancer knowledge, there are several limitations of the study that need to be acknowledged. Firstly, although a multi-channel recruitment approach was chosen to include a variety of cancer patients of different ages and stages, the sample is not representative as participants with high educational levels are overrepresented among both groups. Thus, due to the already high level of education of both groups, the PSG membership may have no longer been so decisive for the knowledge levels of the patients, so the identified positive association between knowledge and peer support membership could have even been more prominent among a representative sample. Further, few young patients participated and only literate, German-speaking patients participated, while patients with a migration background are underrepresented. Therefore, there might be a bias in favour of positive reporting, and the knowledge scores across a representative sample with more balanced groups would probably be worse than in our sample.

Secondly, it is to note that the PSG members in our sample were more likely to be men, of older age, and mainly prostate cancer patients, while among non-members, female breast cancer patients were predominant. Thus, a lack of normally distributed groups due to notably differences in age, gender, and cancer type may have led to slightly biased results. Yet, it can be assumed that the results still depict the reality of these groups and that there is no need for a concern of the results’ validity of the results due to the large sample sizes. Moreover, we did not assess frequency of participation in PSGs, although this may have also been influential on patients’ knowledge. Lastly, we cannot proof causality between PSG participation and cancer-specific knowledge, although it appears plausible that PSG participation increases knowledge.

Lastly, the BCKS-14 does not cover all aspects of knowledge that are shared in PSGs and entails aspects of numeracy knowledge, which is not usually taught in PSGs. It rather focuses on core contents that were identified as central to know by patients. It could be assumed that if a higher number of questions on specific cancer-related knowledge were included on a more extended knowledge scale, the differences between peer support members and non-members would have even been more apparent.

## Conclusion

The results indicate that indeed, PSG members know significantly more about cancer than non-members. PSG membership is a relevant factor contributing to higher knowledge with a small effect, while education is the most decisive predictor for cancer-specific knowledge. Since we found large ranges regarding the knowledge scores among both groups, the findings show the need to inform all patients properly. This could be achieved by giving information material on hand and including relatives during the treatment phase, in order to ensure informed patients. Considering that sociodemographic and clinical variables such as time since diagnosis are also associated with cancer-specific knowledge, cancer care providers should further ensure all patients have access to PSGs early on, especially for patients with lower socioeconomic status. For elderly patients in particular, relatives should be involved and PSGs suggested to improve their knowledge and self-management. Further strategies for improving comprehension and recall could contain prioritising and categorising information, using simple language, and presenting information in different formats, e.g., written, face-to-face, or video information.

## Supplementary Information

Below is the link to the electronic supplementary material.

Supplementary file1 (DOCX 15 KB)

## Data Availability

The datasets used and/or analysed in this study are available from the corresponding author on request.
